# Levels of Plasma Coenzyme Q_10_ Are Associated with Physical Capacity and Cardiovascular Risk in the Elderly

**DOI:** 10.3390/antiox11020279

**Published:** 2022-01-29

**Authors:** Rocío de la Bella-Garzón, Cristina Fernández-Portero, David Alarcón, Josué G. Amián, Guillermo López-Lluch

**Affiliations:** 1 Department of Physiology, Anatomy and Cell Biology, Andalusian Centre of Developmental Biology, Universidad Pablo de Olavide, 41013 Seville, Spain; rdegar1@alumno.upo.es; 2 Department of Social Antropology, Psychology and Public Health, Universidad Pablo de Olavide, 41013 Sevilla, Spain; cbferpor@upo.es (C.F.-P.); dalarub@upo.es (D.A.); jgarami@upo.es (J.G.A.); 3 Centro de Investigación Biomédica en Red de Enfermedades Raras (CIBERER, U729), Instituto de Salud Carlos III-Madrid, Av. Monforte de Lemos, 3-5. Pabellón 11. Planta 0, 28029 Madrid, Spain; 4 Centro de Investigación en Rendimiento Físico y Deportivo, Universidad Pablo de Olavide, 41013 Sevilla, Spain

**Keywords:** Coenzyme Q10, ubiquinone, aging, health, fragility, cholesterol

## Abstract

Coenzyme Q_10_ (CoQ_10_) is an essential factor for mitochondrial activity and antioxidant protection of cells, tissues and plasma lipoproteins. Its deficiency has been associated with aging progression in animals and humans. To determine if CoQ_10_ levels in plasma can be associated with frailty in elderly people (aged > 65), we studied the relationship of CoQ_10_ levels in blood with other parameters in plasma and with the physical activity and capacity in aged people. Our results indicate that high CoQ_10_ levels are directly associated with lower cardiovascular risk measured by the quotient total cholesterol/HDL cholesterol. Furthermore, high CoQ_10_ levels were found in people showing higher physical activity, stronger muscle capacity. CoQ_10_ also showed a strong inverse relationship with sedentarism and the up and go test, which is considered to be a frailty index. Interestingly, we found gender differences, indicating stronger correlations in women than in men. The importance of the maintenance of CoQ_10_ levels in elderly people to avoid sarcopenia and frailty in elderly people is discussed.

## 1. Introduction

Coenzyme Q_10_ (CoQ_10_) is a key component for cell metabolism and antioxidant protection [1]. In mitochondria, CoQ_10_ is located into the inner mitochondrial membrane in which it acts as essential component of the electron transport chain (ETC) [2]. In mitochondria, and in the rest of the membranes, CoQ_10_ is the main antioxidant for the lipidic milieu reducing oxidative damage of phospholipids and controlling by this mechanism cell survival [3,4,5,6]. Furthermore, in plasma lipoproteins, CoQ_10_ is the main protective agent reducing the peroxidation of these proteins, reducing cardiovascular risk by decreasing oxidation of low-density lipoproteins (oxLDLs), especially in elderly people [7]. 

Because of these essential activities, CoQ_10_ can be consider a key component for the physiology of cells that play an important role in the progression of aging [1,8,9]. In fact, decreasing levels of CoQ_10_ have been associated with the dysfunction of several organs in aged people. CoQ is synthesized by a synthome comprising at least 11 different proteins acting between the endoplasmic reticulum and mitochondria [9]. The inability of cells to synthesize CoQ produces many severe symptoms, mainly affecting the cerebellum, the kidneys, and muscle [10]. This indicates the importance of the maintenance of CoQ levels for correct physiological activity in many organs.

In previous studies, we determined that CoQ_10_ levels showed a different behavior in young from that in old people, representing a different physiological response that is probably associated with the levels of oxidative damage [11]. Furthermore, CoQ_10_ levels showed an inverse relationship with obesity and with oxidative damage in plasma [12]. CoQ_10_ can be obtained from the diet although the amount obtained seems to be very low from this source. In blood plasma, CoQ_10_ levels have been studied in aging to determine whether they can be considered to be aging-related biomarkers [9,13]. 

Taking into consideration the essential role of CoQ_10_ in many metabolic and antioxidant activities in the organism, the present work tries to determine whether CoQ_10_ levels in plasma can be associated with frailty risk in elderly people, and if CoQ_10_ can be considered a therapeutic target to reduce sarcopenia and improve functional capacity during aging. 

## 2. Materials and Methods

### 2.1. Study Design and Subjects

We conducted a cross-sectional study. Volunteers were recruited between 1 February and March and tests and sampling were performed until 30 June 2019 from a nursing home and two APC (Active Participation Centers) located in Seville, in the south of Spain. A total of 64 volunteers from three different day centers finally completed the study. The nursing home is a publicly owned center for valid and assisted patients. The medical service of the nursing home was responsible for randomly providing us subjects who voluntarily wanted to participate in the study. At the APC, the study was announced on the bulletin board, and the users accepted participation in the study voluntarily. 

First, all the participants were informed about the objective of the project and the procedures, and signed an informed consent before starting any procedure. All the procedures performed in this study were approved by the Ethic Committee for Biomedical Research of the Andalusian Government with the number 2355-N-19, follow the indications of the International Conference of Good Clinical Practices and were conducted in accordance with the 1975 Declaration of Helsinki guidelines. 

### 2.2. Participants

The criteria for inclusion in the study were being 65 years old or older and being ready to participate in physical tests and in blood extraction. The exclusion criteria were: being under 65 years old, subjects who for medical reasons were contraindicated to perform physical exercise, had suffered congestive heart failure, had suffered around the time of the study joint pain, chest pain, vertigo, or angina, and those who had uncontrolled high blood pressure (160/100). Furthermore, participants receiving therapies for hypercholesterolemia and other diseases needing statin treatments that can affect plasma CoQ_10_ levels were also discarded. 

**Figure 1:**
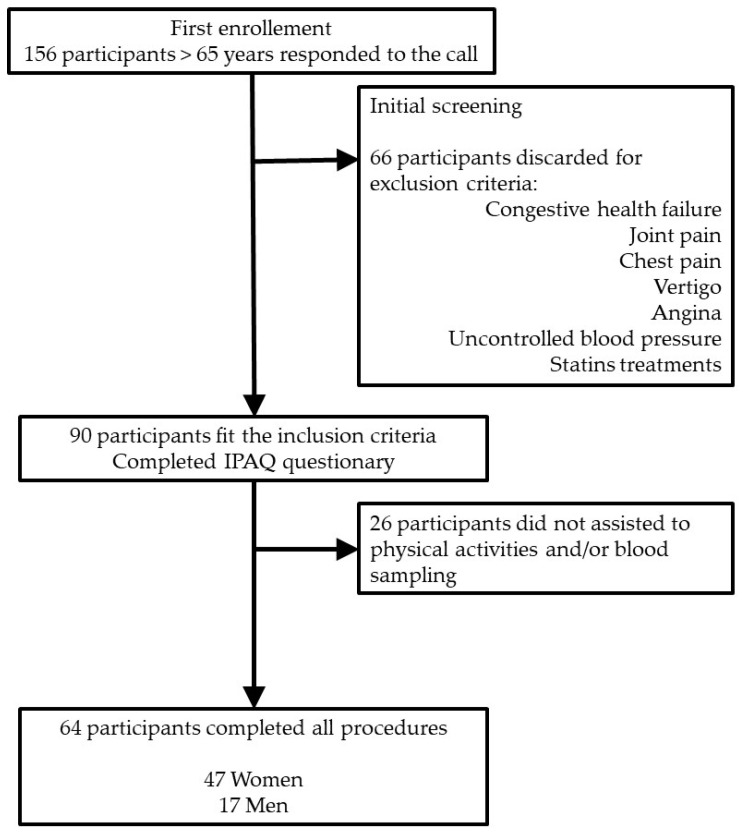
Flow chart of the study selection.

A total of 156 subjects initiated the process of selection. After a first screening in which subjects were asked for their health, diseases and treatments, only 90 of them did not fit the exclusion criteria, and were considered able to perform physical activity tests and blood extraction procedures. During physical activity tests and blood testing, 26 participants did not attend to the session or had problems completing the physical activities. Finally, only 64 participants, 17 men and 47 women, fulfilled all the conditions and participated in all the procedures (Figure 1). Procedures were always carried out in the mornings.

The smoking and moderate drinking habits of the participants are included in Table 1. In general, a significant number of men maintained smoking habits during the study, whereas no woman maintained this habit. Most of the women did not smoke actively, whereas most of the men did. Regarding drinking habits, both men and women maintained moderate drinking habits at the time of the study, with sporadic consumption of beer and wine. 

Most of the participants received treatments against hypertension, mainly enalapril and vasartan, inflammation, such as paracetamol or aspirin, as well as gut protective compounds such as omeprazole. Most of the men received metformin treatment against type II diabetes. In general, these treatments have not been associated with the decrease of CoQ_10_ levels in plasma. Thus, we can consider the clinical conditions of the participants to reflect the normal situation of elderly people in the Spanish population.

### 2.3. Anthropometric Determinations

Anthropometric study was carried out by determining subjects’ height and weight. BMI was estimated by dividing weight (kg) by height squared (m^2^). Bio-electrical impedance (Tanita BF 350) was used to determine total body fat mass, kg of muscle and the percentage of fat, basal calories and metabolic age. Blood pressure was determined by an OMROM monitor. Data from the whole population and separated by gender is indicated in (Table 2).

Metabolic age was calculated comparing the particular basal metabolic rate (BMR) of a participant with the BMR of a chronological age group. The Difference in Age (DIA) is considered the difference between the chronological age and the metabolic age. Both metabolic age and DIA are considered to be parameters that predict individuals that are at high risk of developing metabolic syndrome [14]. 

### 2.4. Determination of the Physical Capacity

The senior fitness battery of tests (SFT) was used to assess major functional fitness components of elderly people [15]. All procedures were carried out in the morning (between 9 and 11 a.m.) in a room with a pleasant temperature for the time of year (23 ± 2 °C). Tests were carried out by same-sex couples, with the exception of those who expressly communicated no problems. Each participant completed six exercise tests in the following order: 30 s chair stand, arm curl, 2 min step, chair sit and reach, back scratch and up and go tests accordingly with the indications previously published [15,16].

The 30 s chair stand test measured functional lower body strength. The arm curl test evaluated functional upper body strength. The 2-min step test (TMST) is one of many alternatives to 6 min walking test for determining exercise capacity [17]. The sit and reach test assessed the flexibility of lower back and hamstring muscles. The back scratch test measured the flexibility of the upper body and shoulders. In addition, the timed up and go test determined the risk of fall [18]. 

### 2.5. Determination of the Level of Physical Activity (IPAQ)

To evaluate the level of physical activity, we used the International Physical Activity Questionnaire (IPAQ) [19]. We used the short, self-administered last-week version of the questionnaire, which includes seven items. The IPAQ considers the time spent being physically active during the last 7 days, including computation of minutes of sitting, walking at moderate intensity, and vigorous-intensity activities. Total METs were calculated as follows: (daily minutes of vigorous walking x days per week practicing walking × 3.3) + (daily minutes of moderate-intensity activity x days practicing moderate-intensity activity × 4.0) + (daily minutes of vigorous activity x days per week practicing vigorous activity × 8.0). Level of physical activity was then classified into three categories, representing active (IPAQ3), moderate activity (IPAQ2) or insufficiently active (IPAQ1) as previously indicated [11,12]. 

### 2.6. Blood Sampling

In the blood sampling session, blood was collected after overnight fasting (10 h) by venipuncture in Vacutainer tubes containing heparin or EDTA as anticoagulants always between 8 and 10 a.m. After centrifuging at 3000× *g* at room temperature, plasma was collected, transferred to several Eppendorf tubes, and stored at −80 °C until measurements as previously indicated [11,12]. 

### 2.7. CoQ_10_ Determination in Plasma 

CoQ_10_ plasma levels were quantified using a protocol described elsewhere [20]. A mixture of ethanol: isopropanol (95:5) was added to 100 μL of blood plasma and mixture vortexed for 1 min. As internal standard to control extraction, 100 pmol of CoQ_6_ were used. After adding 1 mL of hexane, samples were centrifuged at 1000× *g* for 5 min at 4 °C. The upper phases from three consecutive extractions were recovered and dried using a Speed Vac. The dried lipid extract obtained was dissolved in 1 mL of ethanol, dried again in a Speed-Vac, and residue stored at −80 °C until analysis. 

Dried samples were dissolved in the suitable volume of ethanol prior to HPLC injection. Lipid components were separated by a Beckman 166-126 HPLC system equipped with a 15-cm Kromasil C-18 column maintained at 40 °C in a column oven. Separation was performed using a mobile phase containing 65:35 methanol/n-propanol and 1.42 mM lithium perchlorate at aflow rate of 1 mL/min and. CoQ_10_ levels were analyzed with ultraviolet (System Gold 168, Beckman-Coulter, Indianapolis, IN, USA)-based detectors and an electrochemical detector Coulochem III (ESA, Chelmsford, MA, USA). CoQ_10_ content was determined as nmol/mL (μM).

### 2.8. Determination of Other Parameters in Plasma

The lipid profile of subjects (total cholesterol, HDL, and triglycerides), general biochemical profile (glucose, transaminases (glutamate-pyruvate transaminase or alanine transaminase (GPT), gamma-glutamyl transferase (GGT) and glutamyl oxaloacetic transaminase or aspartate transaminase (GOT)), and creatinine, uric acid, urea, and bilirubin), as well as muscle damage (creatine kinase, CK), were determined by using the Reflotron Plus systems (Roche Diagnostics, S.L, Barcelona Spain.). Low-density lipoproteins (LDL) were determined using the Friedewald’s formula [21]. VLDL levels were calculated as triglyceride levels/5. Non-HDL levels were determined as total cholesterol-HDL.

LDL oxidation levels were determined by ELISA by using the Oxidized LDL ELISA kit as indicated by the manufacturer (Mercodia, Sweden).

### 2.9. Statistical Analysis

All results are expressed as mean ± standard deviation (SD). After confirming the normal distribution of the data for all variables, differences between groups were determined using Student’s *t*-test for two groups comparisons or two-way ANOVA with Bonferroni’s post hoc test for comparisons of more than 3 groups. Statistical analysis and Figures were performed by using the GraphPad Prism version 7.00 program (GraphPad Software Inc, San Diego, CA, USA). For correlations, the Pearson’s r analysis was performed. Levels of relationship were determined based on the recommendations of Cohen [22]; an r between 0.1 and 0.29 was considered low, an r between 0.2 and 0.49 was considered moderate, and an r more than 0.5 was considered to be indicative of high correlation. The critical significance level α was established at 0.050 and then, statistical significance was defined as *p* < 0.05. 

## 3. Results

### 3.1. Biochemical Characterization of the Participants

All participants were older than 65, with the group of women being a bit older than the group of men. As expected, gender differences were found between the participants being men significantly taller and heavier than women are. In general, the group showed high BMI in men being a bit lower than in women, but into the overweight range in both cases. Interestingly, although the percentage of fat was higher in women than in men, the amount of visceral fat was higher in men. This indicates a different distribution of the fat in the body that can affect physical capacity. 

Metabolic age of the participants was similar in both groups, although, interestingly, in men the mean of this parameter was higher than the chronological age, whereas in women it was lower. However, in any case, this difference was not significant (Table 2). No differences in blood pressure between the groups were found.

Regarding biochemical parameters in plasma, no differences between men and women were found except in parameters related to muscle damage, such as CK or GGT, which showed significantly lower levels in women in comparison with men. On the other hand, GOT showed an opposite trend. Furthermore, creatinine levels were also higher in men in comparison with women (Table 3). Regarding cardiovascular risk, determined as the quotient between total cholesterol levels and HDL levels > 4 or the levels of non-HDL cholesterol, no differences were found between genders, although in men a tendency to show higher levels was found. In the case of the ratio total cholesterol/HDL > 4, 59% (10/17) of men and 51% (24/47) of women were considered at risk.

**Figure 2:**
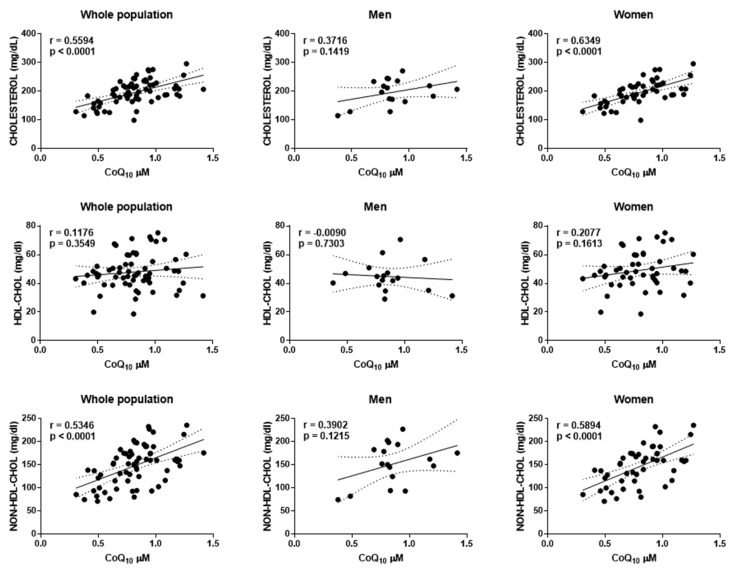
Correlation among plasma CoQ_10_ levels (μM) and total cholesterol, HDL cholesterol and non-HDL cholesterol in plasma. Pearson’s r correlation (r) and statistical significance are indicated.

As expected, plasma CoQ_10_ levels strongly correlated with total cholesterol (Pearson’s r = 0.5594, *p* < 0.0001) and non-HDL cholesterol (Pearson’s r = 0.5346, *p* < 0.001) in the whole population (Figure 2). However, when analyzed separately by gender, in men, this correlation was absent for both total cholesterol (Pearson’s r = 0.3716, *p* = 0.1419) and non-HDLL cholesterol (Pearson’s r = 0.3902, *p* = 0.1215), whereas it was maintained in women with a stronger correlation coefficient, for total cholesterol (Pearson’s r = 0.6349, *p* < 0.001) and non-HDL cholesterol (Pearson’s r = 0.5894, *p* < 0.001). No significant correlation was found with any of the other parameters determined, indicating no clear relationship with other metabolic parameters, even with oxidized LDL cholesterol (Table 4). 

We considered that this lack of significance could be due to the smaller size of the male sample in comparison to the female sample. Unfortunately, we could not increase the size of the sample, since there were no more men that participated in all of the procedures involved in the study. However, in a previous study, another cohort of volunteers aged more than 65 years was also analyzed [11,12], and we calculated the relationship between CoQ_10_ and cholesterol in the blood of this group (unpublished results). In these studies, performed during 2013–2014, the same gender-dependent correlation was found, stronger for women, and lower or null for men. We consider this fact to be very interesting for further experiments, and, at least in elderly people, gender differences in CoQ_10_/cholesterol and related compounds in elderly people must be considered. 

**Figure 3:**
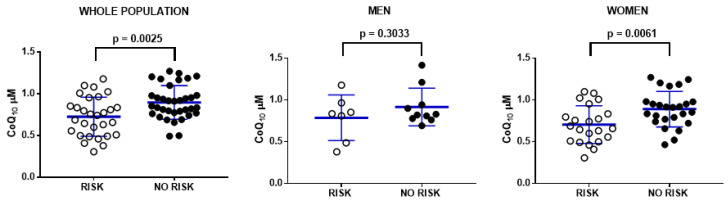
Blood plasma CoQ_10_ levels (μM) in the participants in relationship with their respective cardiovascular risk determined by a total cholesterol/HDL quotient higher than 4 (risk) or lower than or equal to 4 (no risk). Data represent the mean ± SD of the whole population (n = 64), men (n = 17) and women (n = 47).

Some studies have reported that cardiovascular risk can be measured as the quotient between total cholesterol and HDL higher than 4 [23]. Interestingly, when we associated the levels of CoQ_10_ in plasma of the participants depending on their cardiovascular risk, people with low risk showed higher CoQ_10_ levels in plasma than participants with cardiovascular risk. Interestingly, this relationship was not significant in men, probably due to the lower amount of participants, but it was clearly significant in women (Figure 3). 

### 3.2. CoQ_10_ Levels in Plasma Correlates with Physical Activity

Regarding the physical activity of the participants, the range of activity showed a high dispersion. We found participants with a very low activity and others showing higher activity, as determined by METS/week. Interestingly, men showed more active behavior than women in this study. However, when tests from the SFT were performed, no differences between men and women were found, although men showed a tendency to obtain better scores in all the tests except in flexibility. Furthermore, the sitting time was also nearly significantly higher in women than in men (Table 5).

Interestingly, in the whole population, plasma CoQ_10_ levels moderately or strongly correlated with many of the scores of the tests, except in the case of flexibility tests (chair sit-and-reach and back scratch). In men, this correlation disappeared, whereas in women, all the scores showed moderate or high correlation with CoQ_10_ in plasma. In the case of the up and go test, the correlation was negative, since a higher time to return to the chair indicates a lower performance. Thus, this negative correlation indicates that high levels of CoQ_10_ in plasma are associated with lower time to return to chair, and therefore, lower frailty risk (Table 6).

On the other hand, a negative correlation between CoQ_10_ and sitting time was also found. In general, it is clear that higher activity and performance is associated with higher levels of CoQ_10_ in plasma. 

**Figure 4:**
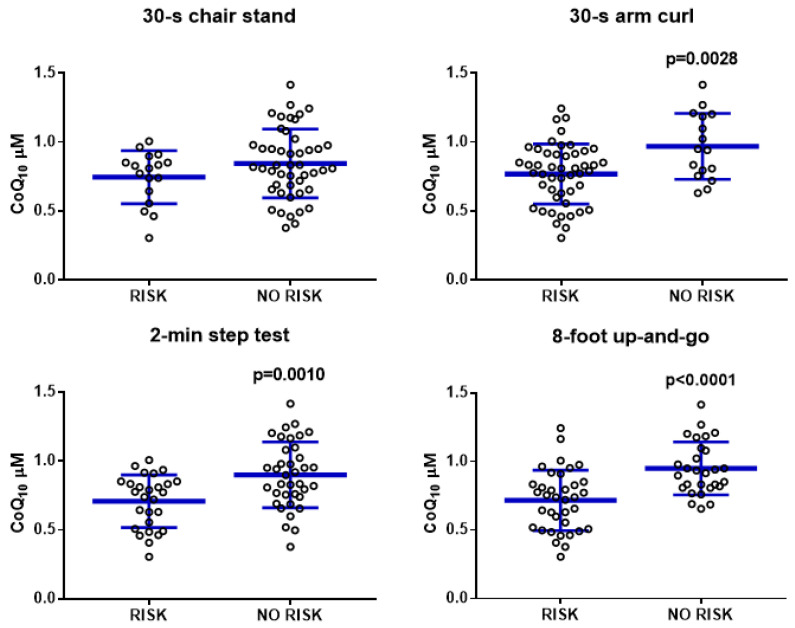
Relationship of blood plasma CoQ10 levels with frailty risk. Blood plasma CoQ_10_ levels (μM) are related to frailty risk, based on the four tests included in the SFT, which showed a direct relationship with CoQ_10_ levels. Data represent the mean ± SD of the whole population (n = 64).

Taking these relationships into consideration, we divided the population according to the risk of frailty depending on the score of the tests of the SFT and determined the levels of CoQ_10_ in plasma [24] (Figure 4). Participants were considered to be at risk of frailty if they scored fewer than eight repetitions for the 30-s chair stand test, fewer than 11 movements for the 30-s arm curl test, fewer than 40 steps for the 2-min step test, or more than 9 s for the up and go test. With respect to all of these procedures, CoQ_10_ levels were higher in the population showing a score indicating low or no risk. This relationship was statistically significant in the case of the 30-s arm curl test, the 2 min step test, and the up and go test (Figure 4). 

**Figure 5:**
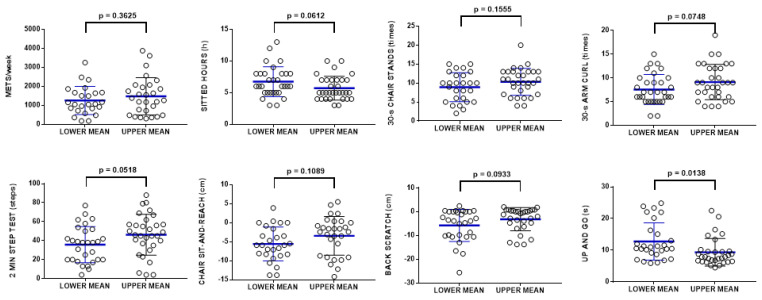
Score of the different test of the SFT depending on the levels of CoQ_10_ in plasma. Mean CoQ_10_ levels were designated as 0.8 μM, and the scores of the participants grouped depending on whether their CoQ_10_ levels in plasma were above or below this mean. Data represent the mean ± SD of the whole population (n = 64).

Furthermore, we also performed an analysis of the score in all the tests included in the SFT with respect to the mean of the levels of CoQ_10_ in plasma. We analyzed the score in the tests of people showing CoQ_10_ levels below the mean in comparison with people above the mean. This analysis indicated that, in general, people showing higher levels of CoQ_10_ in plasma score better in all the tests included in the SFT, although this relationship did not reach statistical significance except in the case of the 8 foot up and go test, in which people showing high CoQ_10_ levels were able to perform this test faster. Furthermore, sedentarism was also lower and near significant in the group showing higher CoQ_10_ levels (Figure 5). 

With respect to the common activity performed by the participants during the week before the procedure (IPAQ), no correlation was found between the levels of CoQ_10_ in plasma and strong or moderate physical activity. However, we did find a moderate correlation between plasma CoQ_10_ levels and intense marching activity (Pearson’s r = 0.364, *p* = 0.031). 

## 4. Discussion

Frailty is a key dysfunction in elderly people that severely affects the capacity of individuals to maintain their independence. Frailty is also associated with comorbidities occurring in aging that impair the health of elderly people [25]. In human skeletal muscle, aging is associated with the accumulation of mitochondrial DNA damage, which contributes to a reduced function of cells and organs. This is reflected in skeletal muscle by changes in the fiber type profile and in the number and size of the fibers in clear relationship with sarcopenia [26]. Other studies demonstrate that mitochondrial function is impaired in skeletal muscle of elderly people at pre-frail condition [27], and that a decrease in the activity of the mitochondrial respiratory chain is associated with lower physical performance and sarcopenia [28]. Moreover, mitochondrial dysfunction, oxidative stress, and DNA damage are associated with the decay in muscle capacity and sarcopenia [29]. It seems now clear that mitochondrial activity and turnover affects many aspects of muscle physiology associated with sarcopenia and the loss of capacity in older individuals [30].

It is known that levels of CoQ_10_ decrease in many tissues and organs during aging [31]. We can then also consider CoQ_10_ levels as a probable factor affecting frailty by accelerating sarcopenia by reducing the activity of mitochondria in muscle. In fact, in aged animals, the amount of CoQ in mitochondria is reduced in skeletal muscle, suggesting a direct relationship of this decrease with the mitochondrial dysfunction associated with aging [31]. 

Furthermore, indirect evidence indicates the importance of CoQ_10_ in the maintenance of physical activity in several diseases, many of them associated with aging [2]. In animal studies, CoQ_10_ supplementation improved plasma parameters associated with acute exercise such as lactate, ammonium and CK, and also increased liver and muscle glycogen content, improving exercise performance and reducing fatigue [32]. Additionally, animal studies have also shown a clear decrease of CoQ levels in muscle in aged individuals [33]. Supplementation with CoQ_10_ prevented mitochondrial dysfunction in muscle [34], indicating a clear role of CoQ_10_ in mitochondrial activity and probably in muscle capacity during aging. 

In humans, patients suffering osteoarthritis and healthy elderly people showed a negative significant correlation between oxidative stress and muscle function [35]. In this study, plasma CoQ_10_ levels were positively associated with antioxidant capacity, muscle mass, strength and endurance in these patients, indicating a relationship with physical capacity. On the other hand, the amount of CoQ_10_ is frequently reduced in muscle of patients suffering mitochondrial myopathies, and supplementation has been suggested to improve motor function [36]. Furthermore, CoQ_10_ levels in plasma have been associated with the control of motor activity in multiple system atrophy patients. In these patients, lower CoQ_10_ levels in plasma are associated with higher severity of motor control dysfunction [37]. 

Our results agree with the relationship of plasma CoQ_10_ levels and pathological conditions. In agreement with cardiovascular studies such as Q-SYMBIO [38,39,40], we found that high CoQ_10_ levels are associated with lower cardiovascular disease risk. This reinforces the evidence indicating the thereapeutic use of CoQ_10_ in the prevention of cardiovascular diseases [41]. The relationship of high CoQ_10_ with higher physical activity is also related to better cardiovascular health [42], and heightens the importance of this compound in the function of the cardiovascular system and the protection of endothelial vasculature. 

On the other hand, although muscle cannot incorporate plasma CoQ_10_ easily [43], vascular endothelium does incorporate this compound [44]. Therefore, we cannot discard a direct role of high plasma CoQ_10_ levels in the endothelial function of arteries feeding skeletal muscle [45]. High CoQ_10_ levels could maintain the mitochondrial activity of the vasculature, and by this mechanism improve muscle capacity. In fact, long-term CoQ_10_ supplementation in rats improves endothelial function in aged rats [46]. In humans, mitochondrial antioxidants such as CoQ_10_ improve endothelial function and the activity of antioxidant enzymes increasing exercise tolerance in patients with peripheral artery diseases [47]. These results suggest that the maintenance of high levels of plasma CoQ_10_ in active people contributes to the protection of the vascular endothelium against oxidative damage, preventing cardiovascular diseases such atherosclerosis by this mechanism. 

Regarding physical performance, our results are in agreement with previous studies demonstrating a positive correlation between CoQ_10_/cholesterol levels and hand grip capacity in elderly people [48]. Furthermore, in the Fisher’s group study, low levels of the reduced form of CoQ_10_ were associated with lower muscle capacity, indicating that low levels of CoQ_10_ can be considered a plasma indicator of higher risk of sarcopenia [48]. Our results also indicate that high levels of plasma CoQ_10_ are associated with lower risk for suffering muscle dysfunction, reinforcing the relationship between plasma CoQ_10_ levels and muscle capacity. 

Our previous studies also demonstrated that high physical activity is associated with higher CoQ_10_ levels in plasma and lower levels of lipid peroxidation, increasing antioxidant protection [12]. Furthermore, sedentarism and obesity are associated with lower CoQ_10_ levels [12], which can also aggravate the oxidative damage produced by higher resting and exercise-induced intramuscular free radical release in aged muscle [49]. Furthermore, we recently demonstrated that in professional soccer players, high CoQ_10_ levels are associated with low stress and damage markers and with higher performance during competition [50].

Animal studies have also demonstrated that supplementation with CoQ_10_ improves antioxidant capacity, especially in aged animals [51], reducing lipid peroxidation damage [52]. Furthermore, exercise can induce CoQ-dependent antioxidant activities in aged muscle, preventing oxidative damage [53]. This could be the reason the combination of moderate physical activity with CoQ_10_ supplementation counteracts mitochondrial dysfunction in the accelerated aging SAMP8 mouse model [54]. Accordingly, in humans, the combination of exercise with CoQ_10_ has been suggested to counteract sarcopenia in the elderly [55]. For this reason, CoQ_10_ supplementation has been be suggested to decrease many dysfunctions associated with aging and age-related diseases [9,56,57].

Some important aspects remain to be studied. The different behavior of the ratio CoQ_10_/cholesterol in men and women need further research to confirm this fact, at least in elderly people. On the other hand, it has recently been shown that older adults suffering physical frailty and sarcopenia show a release of small extracellular vesicles with mitochondrial signatures [58]. The presence of these vesicles is associated with the senescence secretory phenotype associated with aging [59]. To date, few articles have studied the role of CoQ_10_ in the evolution of this characteristic of aging, although none of them have been studied in muscle. Supplementation with CoQ_10_ reduces senescence phenotype in culture fibroblasts [60]. Interestingly, CoQ_10_ protects endothelial cells against inflammatory response and the secretory phenotype preventing the release of IL-6 [61], a known cytokine that affects muscle sarcopenia [62]. The effect of CoQ_10_ has been directly related with its antioxidant activity [63]. 

Our study shows correlations between CoQ_10_ levels in plasma and physical activity in elderly people. Obviously, its main limitation is the lack of correlation of CoQ_10_ levels in muscle with its capacity, but due to obvious ethical aspects, this determination cannot be performed. However, our results and many other studies suggest that high plasma CoQ_10_ levels could be associated with the maintenance of physical capacity in elderly people. It is probable that supplementation with CoQ_10_ could improve physical capacity in addition to the known effects in the cardiovascular system. These relationships suggest that CoQ_10_ could be considered an important component for maintaining independence and health in aged individuals.

Furthermore, due to the difference in the number of men and women that participated in this study, gender differences must be considered with caution. Nevertheless, previous studies by our group also found gender differences in plasma [12]. Our results suggest that gender may be an important factor in CoQ_10_ distribution in plasma, and studies must consider this fact in order to clarify whether these differences are relevant for aging and age-related diseases [64]. Interestingly, recent studies have also demonstrated a gender-dependent bioavailability in response to a single dose of CoQ_10_ supplementation. This aspect must also be considered in further studies [65].

## 5. Conclusions

It is clear that maintenance of active physical capacity during aging is essential for avoiding frailty [66]. Many studies support the positive effect of CoQ_10_ on physical activity. Combination of CoQ_10_ with physical activity could be an important therapy for avoiding sarcopenia and maintaining higher capacity during aging. Importantly, gender differences found in this study must be studied in depth in order to determine whether the effect of CoQ_10_ is stronger in women than in men and to design therapeutic strategies in accordance with these differences. Moreover, the relationship between CoQ_10_ and cholesterol found in elderly people will be a subject for future works in order to determine whether age is a factor in the correlation between cholesterol and CoQ_10_ in plasma. Furthermore, the association of physical activity in elderly people with high CoQ_10_ levels could not only improve endothelial capacity, but also reduce the release of vesicles and factors involved in inflammation and the senescence phenotype. Further studies are needed to determine whether CoQ_10_ can also reduce this phenotype in muscle, since in the case of positive relationship CoQ_10_ could be considered a senolitic compound for muscle.

## Figures and Tables

**Table 1:** Age, social and clinical characteristics of the participants in the study.

Parameter	Total Population (n = 64)	MEN (n = 17)	WOMEN (n = 47)
	Mean ± SD	Mean ± SD	Mean ± SD
Age			
From 65 to 75	32 (50.0%)	11 (64.7%)	21 (44.68%)
More than 76	32 (50.0%)	6 (35.3%)	26 (55.32%)
Pharmacological treatments			
	59 (92.2%)	16 (94.1%)	43 (91.5%)
Hypertension	30 (46.9%)	10 (58.8%)	20 (42.6%)
Diabetes	8 (12.5%)	5 (29.4%)	3 (6.4%)
Others	47 (73.4%)	11 (64.7%)	35 (74.5%)
Smoking habits			
Never	38 (59.3%)	1 (5.9%)	37 (78.7%)
Before	20 (31.3%)	10 (58.8%)	10 (21.3%)
During study	6 (9.4%)	6 (35.3%)	0 (0.0%)
Drinking habits			
Never	19 (29.7%)	0 (0.0%)	19 (40.4%)
Before	13 (20.31%)	9 (52.9%)	4 (2.5%)
During study	32 (50.0%)	8 (47.1%)	24 (51.1%)

**Table 2:** Characteristics of the participants in the study.

Parameter	Total Population (n = 64)		MEN (n = 17)		WOMEN (n = 47)		Significance
	Mean ± SD	Range	Mean ± SD	Range	Mean ± SD	Range	*p*
Age (y)	77.0 ± 8.0	65–99	73.4 ± 6.3	65–84	78.3 ± 8.2	66–90	0.0381 *
Metabolic age (y)	76.5 ± 11.0	48–90	76.4 ± 13.1	48–90	76.5 ± 10.2	55–90	0.9803
DIA (y)	0.5 ± 12.6	−20–30	−3 ± 16.1	−20–30	1.8 ± 11.0	−15–15	0.1847
BP Systolic	134 ± 17	89–167	134 ± 17	89–164	134 ± 17.6	94–167	0.9617
BP Diastolic	72.9± 9.6	48–93	70.4 ± 10.6	48–93	74.8± 12.4	52–132	0.1976
Height (cm)	154.1 ± 7.4	138–173	160.6 ± 5.6	149–173	151.8 ± 6.6	138–167	<0.0001 *
Weigth (kg)	70.6 ± 8.6	49.2–90.7	74.2 ± 7.9	60.0–88.4	69.2 ± 8.6	49.2–90.7	0.0426 *
BMI	29.8 ± 3.9	22.6–40.8	28.7 ± 3.1	23.4–35.5	30.2 ± 4.5	19.9–40.9	0.2277
Fat (%)	40.4 ± 7.8	21.7–61.7	32.5 ± 5.4	21.7–40.1	43.3 ± 6.4	24.1–61.7	<0.0001 *
Muscle (kg)	39.7 ± 6.6	25–61	47.4 ± 5.5	38.4–61	36.9 ± 4.5	25–49.6	<0.0001 *
Visceral fat (kg)	14.7 ± 4.0	7–30.4	18.8 ± 4.2	12–30.4	13.2 ± 2.3	7–19	<0.0001 *

Data represent the mean ± SD of each parameter and the range in the population. DIA = Difference in age; BP: Blood Pressure; BMI: Body Mass Index. * Significant differences between gender are indicated for each parameter, SD: standard deviation.

**Table 3:** Blood plasma biochemical parameters of the participants in this study.

Parameter	Total Population (n = 64)		MEN (n = 17)		WOMEN (n = 47)		Significance
	Mean ± SD	Range	Mean ± SD	Range	Mean ± SD	Range	*p*
Total CoQ_10_ (μM)	0.818 ± 0.238	0.306–1.416	0.862 ± 0.247	0.378–1.416	0.806 ± 0.236	0.306–1.269	0.3859
Chol (mg/dL)	196.1 ± 42.9	99–296	197.0 ± 45.1	115–271	195.8 ± 42.6	99–296	0.9214
HDL-chol (mg/dL)	48.0 ± 12.3	18.6–75.3	44.8 ± 10.7	29–70.6	49.1 ± 12.8	18.6–75.3	0.2166
LDL-chol (mg/dL)	128 ± 42	51–215	132 ± 46	58.1–205	126.5 ± 40.9	51–215	0.6451
VLDL-chol (mg/dL)	20.2 ± 6.7	14–41	20.2 ± 6.9	14–36	20.2 ± 6.6	14–41	0.9931
Non-HDL-chol (mg/dL)	148 ± 42.2	71.1–236	152 ± 45.4	74.7–227.3	146 ± 41.4	71.1–235	0.6458
Chol/HDL-chol	4.3 ± 1.3	2.3–7.9	4.6 ± 1.3	2.3–6.6	4.2 ± 1.3	2.3–7.9	0.3239
TGs (mg/dL)	100.8 ± 33.3	69.9–205	100.9 ± 34.6	69.9–180	100.8 ± 33.2	69.9–205	0.9917
CoQ_10_/Chol (nmol/mmol)	163.2 ± 44.5	85.6–316.3	172.7 ± 50.3	114–264	159.8 ± 42.2	85.6–316	0.3059
CoQ_10_/HDL-chol (nmol/mmol/	705 ± 303	273–1746	791 ± 346	363–1746	674 ± 284	273–1684	0.1744
CoQ_10_/LDL-chol (nmol/mmol)	265 ± 92.6	127–538	275 ± 105	158–538	261 ± 88.5	127–538	0.5821
CoQ_10_/VLDL-chol (nmol/mmol)	1690 ± 638	596–3219	1773 ± 644	881–2990	1661 ± 640	596–3219	0.5383
CoQ_10_/non-HDL-chol (nmol/mmol)	222 ± 67.4	113.8–398.7	231 ± 75.4	145–399	219 ± 64.8	114–390	0.5436
oxLDL (U/L)	75.6 ± 17.1	34.6–105	77.8 ± 19.5	34.6–109	75.5 ± 16.4	46.4–105	0.6316
CK (U/L)	69.8 ± 36.8	24.3–226.0	86.0 ± 49.6	24.3–226.0	64.0 ± 29.4	24.3–159	0.0335 *
GGT (U/L)	18.1 ± 23.4	0.49–144	29.1 ± 36.1	0.5–144	13.8 ± 15.1	0.5–82.4	0.0196 *
GOT (U/L)	21.2 ± 6.5	5–37.4	18.0 ± 6.7	5–29.2	22.4 ± 6.0	12–37.4	0.0153 *
GPT (U/L)	15.1 ± 5.1	6.5–32.8	16.7 ± 5.6	6.5–25.2	14.5 ± 4.8	7.5–32.8	0.122
BILIRRUBIN (mg/dL)	0.76 ± 0.19	0.49–1.28	0.72 ± 0.20	0.49–1.28	0.78 ± 0.19	0.5–1.3	0.3505
CREATININE (mg/dL)	1.16 ± 0.28	0.63–2.18	1.28 ± 0.43	0.49–2.18	1.09 ± 0.22	0.49–1.86	0.0302 *
URIC ACID (mg/dL)	5.75 ± 1.75	1.99–10.1	6.25 ± 1.79	3.23–9.70	5.57 ± 1.71	1.99–10.1	0.1719
UREA (mg/dL	35.5 ± 14.2	19–87.5	34.1 ± 15.7	19–76.5	36.0 ± 13.7	19–87.5	0.6314
GLUCOSE (mg/dL)	113.6 ± 34.8	38.5–218	123.2 ± 46.1	48.9–218	110.2 ± 29.5	38.5–205	0.189

Data represent the mean ± SD for each parameter. * Significant differences between genders are indicated. Chol: cholesterol; HDL-chol: high density lipoprotein-cholesterol; LDL-chol: low density lipoprotein-cholesterol; VLDL-chol: very low density lipoprotein-cholesterol, TG: triglycerides; oxLDL:oxidated LDL; CK: creatine kinase; GPT: glutamate-pyruvate transaminase or alanine transaminase; GGT: gamma-glutamyl transferase; GOT: glutamyl oxaloacetic transaminase or aspartate transaminase.

**Table 4:** Correlation between CoQ_10_ levels and blood plasma biochemical parameters.

Parameter	Total Population (n = 64)		MEN (n = 17)		WOMEN (n = 47)	
	Pearson r	*p*	Pearson r	*p*	Pearson r	*p*
Chol (mg/dL)	0.56	<0.0001	0.372	0.1419	0.635	<0.0001
HDL-chol (mg/dL)	0.118	0.3549	−0.09	0.7303	0.208	0.1613
LDL-chol (mg/dL)	0.509	<0.0001	0.347	0.172	0.571	<0.0001
VLDL.chol (mg/dL)	0.172	0.1725	0.253	0.3266	0.156	0.2942
Non-HDL-chol (mg/dL)	0.535	<0.0001	0.39	0.1215	0.589	<0.0001
TC/HDL-chol	0.328	<0.0080	0.466	0.0593	0.265	0.0719
oxLDL (U/L)	0.073	0.5642	−0.005	0.9856	−0.001	0.9953
TGs (mg/dL)	0.182	0.1495	0.253	0.3266	0.157	0.2934
CK (U/L)	−0.009	0.9444	0.000	0.9995	−0.066	0.6586
GGT (U/L)	−0.006	0.9634	0.001	0.9693	−0.089	0.5517
GOT (U/L)	0.18	0.154	0.331	0.1937	0.18	0.154
GPT (U/L)	0.128	0.3131	0.110	0.6738	0.109	0.4662
BILIRRUBIN (mg/dL)	−0.009	0.9444	−0.065	0.8045	0.158	0.2876
CREATININE (mg/dL)	−0.013	0.313	0.164	0.5295	−0.095	0.5259
URIC ACID (mg/dL)	−0.058	0.6466	0.031	0.9057	−0.121	0.4172
UREA (mg/Dl	0.085	0.505	0.397	0.115	−0.035	0.8153
GLUCOSE (mg/dL)	−0.143	0.2589	−0.199	0.4433	−0.15	0.3143

Chol: cholesterol; HDL-chol: high density lipoprotein-cholesterol; LDL-chol: low density lipoprotein-cholesterol; VLDL-chol: very low density lipoprotein-cholesterol, TG: triglycerides; oxLDL: oxidated LDL; CK: creatine kinase; GPT: glutamate-pyruvate transaminase or alanine transaminase; GGT: gamma-glutamyl transferase; GOT: glutamyl oxaloacetic transaminase or aspartate transaminase.

**Table 5:** Physical activity parameters.

Parameter	Total Population (n = 64)		MEN (n = 17)		WOMEN (n = 47)		Significance
	Mean ± SD	Range	Mean ± SD	Range	Mean ± SD	Range	*p*
METS/week	1374 ± 874	180–3879	2129 ± 857	918–3879	1103 ± 714	180–3252	<0.0001 *
30 s CHAIR STAND (times)	9.67 ± 3.72	2–20	10.57 ± 3.78	4–15	9.39 ± 3.70	2–20	0.3033
30 s ARM CURL (times)	8.34 ± 3.51	2–19	8.47 ± 3.20	4–15	8.30 ± 3.65	2–19	0.8636
2 MIN STEP TEST (steps)	41.2 ± 20.9	4–88	48.0 ± 27.0	4–88	39.0 ± 18.3	4–79	0.1492
CHAIR SIT-AND-REACH (cm)	−4.48 ± 6.89	−14.2–5.5	−3.70 ± 5.09	−12.2–3.9	−4.68 ± 4.87	−14.2–5.5	0.5403
BACK SCRATCH (cm)	−4.43 ± 5.96	−25.6–2.4	−2.85 ± 4.31	−13–1.8	−4.82 ± 6.31	−25.6–2.4	0.2806
UP AND GO (s)	10.9 ± 5.4	4.5–24.9	10.3 ± 5.7	5.2–22.5	11.2 ± 5.3	4.5–24.9	0.5796
SITTING TIME (h)	6.22 ± 2.17	3–13	5.35 ± 1.84	3–10	6.54 ± 2.22	3–13	0.0529

Data represent the mean ± SD for each parameter. * Significant differences between genders are indicated.

**Table 6:** Correlation between blood plasma CoQ_10_ and scores in the SFT.

Parameter	Total Population (n = 64)		MEN (n = 17)		WOMEN (n = 47)	
	Pearson r	*p*	Pearson r	*p*	Pearson r	*p*
METS/week	0.148	0.2902	0.523	0.0551	−0.023	0.8883
30 s CHAIR STAND (times)	0.372	0.0041	0.191	0.5138	0.344	0.02
30 s ARM CURL (times)	0.45	0.0002	0.344	0.1766	0.428	0.0027
2 MIN STEP TEST (steps)	0.473	0.0001	0.416	0.1233	0.451	0.0019
CHAIR SIT-AND-REACH (cm)	0.214	0.114	−0.079	0.8087	0.325	0.0314
BACK SCRATCH (cm)	0.128	0.3334	0.1628	0.5782	0.158	0.2929
UP AND GO (s)	−0.513	<0.0001	−0.363	0.1838	−0.514	0.0004
SITTING TIME (h)	−0.405	0.001	−0.157	0.5473	−0.399	0.006

## Data Availability

All of the data is contained within the article.
